# Drivers of physical connectivity between coral reefs along the Southeast African coastline

**DOI:** 10.1038/s41598-025-07776-y

**Published:** 2025-07-01

**Authors:** Vibhav Atish Deoraj, Justin James Pringle, Derek Dewey Stretch

**Affiliations:** Environmental Fluid Mechanics Lab, University of KwaZulu-Natal, Durban, 4051 South Africa

**Keywords:** Connectivity, Hydrodynamics, Agulhas, Physical oceanography, Marine biology

## Abstract

The resilience and persistence of coral reef metapopulations strongly depend on their dispersal potential. Larval dispersal influences the diversity and genetic structure of coral populations and contributes to population recovery following disturbances. We assessed the connectivity of coral reefs in the Western Indian Ocean (WIO) during peak spawning periods between 1994 and 2014. The study focused on a broadcast coral, *Acropora austera*, which has a short pelagic larval duration (PLD). High-velocity streams offshore of the Delagoa Bight connect distant reef complexes on the Southeast African coastline. Complex interactions between regional ocean currents and the African continent drive their formation. These regional flow patterns are part of the larger Agulhas Current system, facilitating inter-reef connectivity within the virtual larvae’s PLD due to high current speeds. The evolutionary connectivity of short-lifespan corals identified between Mozambican and South African reefs is also regulated by intermittent regional flow patterns.

## Introduction

The resilience and persistence of coral reef metapopulations strongly depend on their dispersal potential^1^. Larval dispersal influences the diversity and genetic structure of coral populations and contributes to population recovery following disturbances^2,3^. Understanding regional dispersal patterns and connectivity is vital for reef conservation, particularly to identify and protect larval source reefs that support neighbouring reefs^4^.

Due to the limited swimming ability of coral larvae, dispersal during the pelagic phase is controlled primarily by the magnitude and direction of regional ocean currents^2^. Secondary ecological influences during the pelagic phase include buoyancy, development rate, vertical migration, mortality, food availability, and physiological tolerances^5–7^. In the benthic phase, coral larvae use a diverse array of cues to select a suitable settlement habitat, including salinity, light, turbulence, sound, chemical compounds, and oxygen saturation^8^.

The Maputaland Reef Complex (MRC), a World Heritage Site along the Southern African coast, represents the southernmost distribution of African coral reefs, located as far south as 28°S^9,10^. Although they are non-accretionary, the MRC reefs are rich in biodiversity, including both hard and soft coral diversity^10–12^. Notably, coral reefs in this region are considered marginal and exist within suboptimal conditions or at their environmental limits^13^. MRC reefs are located in the Western Indian Ocean (WIO), a region characterised by high mesoscale eddy kinetic energy that interacts with the complex shelf topography to form complex flow fields^14^. Macdonald et al.^15^ highlighted the crucial role of regional flow patterns within the WIO in regulating the degree of reef connectivity, particularly the Agulhas Current that flows southward at speeds of more than 1.5 m/s. Lamont et al.^16^ investigated the hydrodynamics of the Delagoa Bight in Mozambique, observing cyclonic eddies that entrained a surface drifter for up to six weeks. Importantly, few studies have investigated the regional flow patterns that might explain the gene flow findings from field studies on Southern African coral reefs^15,17^. High-latitude reefs that rely on low-latitude reefs for larval supply are at risk of losing that supply given the vulnerability of low-latitude coral reefs to future thermal stresses^18^. Therefore, understanding the extent of connectivity is crucial for conservation efforts in the region.

The coral communities of the MRC have been studied for several decades^12,19–22^. To date, MRC reefs appear to have been relatively unaffected by rising ocean temperatures and have shown limited bleaching^12,20^. However, a 2017 study reported a gradual shift in community structure over twenty years as soft coral cover declined while hard coral cover was generally maintained or increased^12^. An earlier study on the community structure of the Sodwana Bay reefs by Celliers and Schleyer^9^ suggested that the coral communities in the central reef complex were so dissimilar that the protection of any single reef would not protect regional diversity. Macdonald et al.^15^ identified a genetic discontinuity between Mozambican and South African coral reefs, while Montoya-Maya et al.^17^ suggested that there is limited ecologically relevant connectivity between the aforementioned reefs, and that South African reefs exhibit greater biodiversity. These indications of natural variation and change, as well as some degree of isolation of the MRC, reinforce the need for an improved understanding of connectivity in the region.

A well-established approach to assess reef connectivity involves the sampling and genetic analysis of numerous coral species to estimate gene flow between populations^23–25^. However, these studies are resource intensive, particularly on a regional scale where researchers must consider cross-border reef connectivity. Numerical simulations of larval dispersal have gained traction as a viable alternative to genetic analyses due to the efficiency of the method, with numerous studies carried out globally^26–28^. Simulations involve coupling virtual larvae as Lagrangian particles with the output of a regional ocean model to investigate potential larval pathways. The findings of particle tracking studies also help to identify strong source reefs, optimising the efficiency of future field studies.

In this study, we assess the contribution of regional-scale hydrodynamics to coral reef connectivity in the region using global ocean models and particle tracking methods. *Acropora austera*, an ecologically important species of branching coral, was modelled in simulations to provide biological limits for numerical simulations, such as larval competency and lifespan.

## Methods

### Hydrodynamic database

The Global Ocean Physics Reanalysis (GLORYS12V1) produced by the Copernicus Marine Environment Monitoring System (CMEMS) provided daily-averaged horizontal ocean current components over the simulation period. Currents are output on a 1/12° horizontal grid resolution on 50 vertical levels with increased resolution near the surface^29^. The CMEMS model component uses the Nucleus for European Modelling of the Ocean (NEMO) platform forced at the surface by the European Centre for Medium-Range Weather Forecasts (ECMWF) ERA-Interim and ERA5 reanalyses for older and more recent models respectively. The model does not include tidal forcings. Data availability covers 31 years from 1993 to 2024. Observations are assimilated using a reduced-order Kalman filter that includes an adaptive-error estimate and localisation algorithm. Observation datasets include reprocessed sea level anomalies (SLA) from along-track satellite altimeter missions, satellite sea surface temperature from NOAA, and in situ temperature and salinity vertical profiles from the CORA database^29^. The CORA database also includes temperature and salinity vertical profiles from the sea mammal database^30^. The bathymetry used in the model consists of ETOPO1 for deep ocean regions and GEBCO8 along coastlines and continental shelves. Reanalysis currents modelled by the NEMO model have been validated against observations from drogue-only subsurface drifters between 2003 and 2016^31^. The NEMO model has been validated by several studies^28,32–36^. Furthermore, the GLORYS12V1 dataset has been applied to various hydrodynamic studies in the Western Indian Ocean (WIO) at various scales^14,37,38^. Momin^32^ validated the performance of the NEMO model against satellite altimetry in the Indian Ocean. It was therefore concluded that this dataset was appropriate for carrying out this study at a regional scale.

### Particle tracking

The open-source, Lagrangian particle tracking software package OpenDrift^39^ was used to simulate the dispersal of virtual coral larvae. The software provides a framework for simulating the trajectory of various scenarios such as oil spills, icebergs, microplastics, larval dispersal, and search and rescue^40–43^. The package uses a second-order Runge-Kutta solver to propagate particles in time^39^.

A spatially and temporally constant diffusivity or random walk of 933 m$^{2}$/s was applied for all particle tracking simulations to account for sub-grid scale processes not resolved by the computational mesh as well as numerical smoothing in the reanalysis dataset. The diffusivity was calculated as the ratio of the square of the grid size of the global hindcast model (1/12°) divided by the data output frequency of one day. The diffusivity that is used to account for numerical smoothing should not be the dominant diffusion term, and should therefore not exceed the eddy diffusivity used in the NEMO model^38^. The NEMO model has a horizontal diffusive length of 200 km and a horizontal diffusive velocity of 0.01 m/s, resulting in a horizontal eddy diffusivity of 2000 m$^{2}$/s. This implies that the diffusivity from the NEMO model will dominate the numerical smoothing diffusivity used in the Lagrangian model^38^. Particle tracking simulations were performed using an internal time step of 30 minutes. Virtual larvae were assumed to be positively buoyant and hence confined to the surface; therefore no vertical current velocities were used in the simulations. OpenDrift integrates a high-resolution coastline to perform checks within the coastline interaction module. This process mimics a high-resolution coastline regardless of the spatial resolution of the hydrodynamic model.

### Coral reefs

**Fig. 1:**
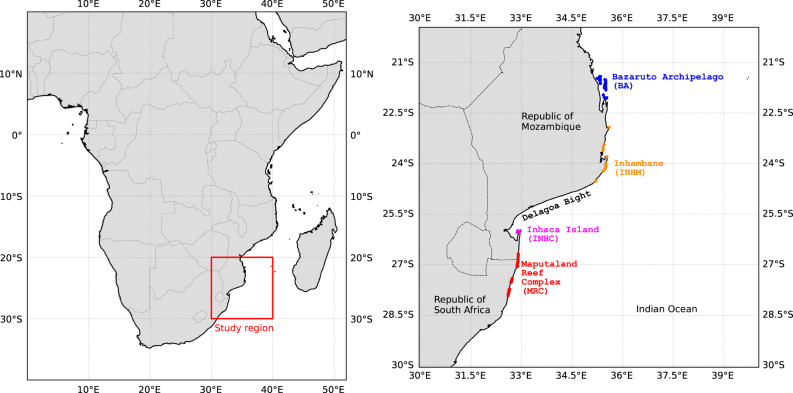
Locality of study region (*left*) and digitised coral reefs used for the connectivity analysis (*right*). The spatial distribution of individual reef complexes are presented using coloured polygons that represent the Bazaruto Archipelago (BA) (*blue*), Inhambane (INHM) (*orange*), Inhaca Island (INHC) (*magenta*) and the Maputaland Reef Complex (MRC) (*red*).

The Global Distribution of Coral Reefs dataset provided by the UNEP World Conservation Monitoring Centre (UNEP-WCMC) was used to identify coral reefs within the study region (^44^; https://data.unep-wcmc.org/datasets/1). Examination of this dataset against reef distribution maps from previous studies showed that UNEP-WCMC omitted some higher-latitude reefs, particularly within the MRC. For these missing reefs, polygons were digitised into shapefiles using QGIS and added to the dataset manually. Figure 1 presents the study region spanning Mozambique and South Africa in the WIO. Four major reef complexes were examined. These include, from north to south, the Bazaruto Archipelago (BA), Inhambane (INHM), Inhaca Island (INHC) and the Maputaland Reef Complex (MRC).

### Virtual larvae assumptions

The coral species represented in the virtual larval tracking was *Acropora austera*, an abundant and ecologically important species in eastern Africa and the only branching coral species that form large monospecific stands with high structural complexity on South African reefs^45–47^. *A. austera*, like most scleractinian coral species in the study area, is a hermaphroditic broadcast spawner, which means that colonies release sperm and egg bundles that float to the surface where they fertilise and develop into larvae^48^. The lipid-rich larvae are buoyant and remain at the surface^49^. Hence, the larval tracking simulations used surface currents only, in agreement with several similar studies exploring physical connectivity^26–28^. Coral connectivity was assessed during the peak coral spawning period in the WIO (February to March)^50,51^. Virtual larvae were released every 2 days throughout February and March for 20 years from 1994 to 2014. The release occurred at midnight to approximate the observed behaviour of broadcast spawners in nature^52^. The number of larvae released was based on the plan area of individual reef polygons. Polygons exceeding 1 km$^2$ were seeded with 200 larvae per km$^2$, while polygons below this threshold were seeded with 200 larvae. This resulted in an initial release of approximately 11,000 particles per simulation. Each virtual larva was tracked at 30-minute intervals for 10 days, after which it was considered inactive. The first four days represented a ‘precompetency’ developmental period during which no settlement occurred, leaving a six-day window for settlement. These temporal limits were based on published laboratory studies on the larvae of *A. austera* and other *Acropora*^53,54^. The limits were set conservatively because a small proportion of coral larvae in laboratory studies survive considerably longer^2,53–55^. During dispersal, a connectivity event was defined as the intersection of a virtual larva with a coral reef polygon. Individual larvae were allowed to connect to multiple destination reefs within their lifespan. Larvae transported to the shoreline accumulated there, but remained active and able to move seaward again if currents changed.

## Results

### Regional inter-reef connectivity

**Fig. 2:**
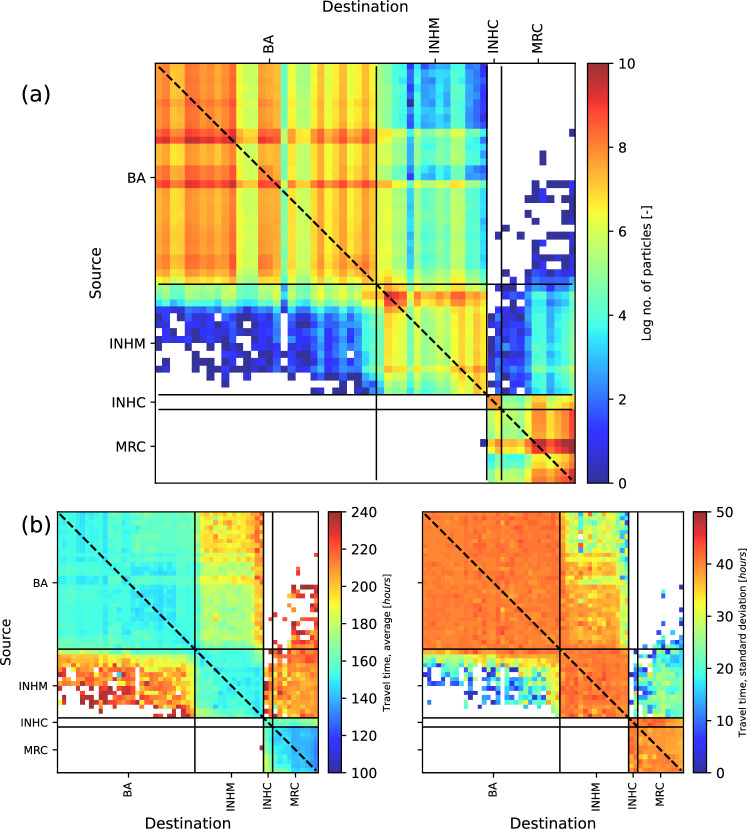
Plot showing (**a**) an inter-reef connectivity matrix for the simulated 20-year period presenting the number of larvae released from source reef *i* reaching destination reef *j* and (**b**) matrices of mean (*left*) and standard deviation (*right*) travel time for virtual larvae reaching destination reefs. Blank regions indicate that no connections were observed.

Figure 2 presents a regional inter-reef connectivity matrix showing the strength of links between reef pairs using the larval properties of *A. austera* for the simulated period. Each element in the matrix indicates the number of larvae released at source reef *i* reaching destination reef *j*. The connectivity located along the diagonal ($i = j$), plotted as the black dashed line in Figure 2, indicates the level of self-recruitment for the source reefs. Individual reef complexes are distinguished using black lines. Source reefs (*left axis*) increase in latitude from top to bottom, while the destination reefs (*bottom axis*) increase in latitude from left to right.

Figure 2a indicates that the highest levels of connectivity occur between reefs belonging to the same complex. This pattern is evidenced by higher levels of connectivity generally falling within the bounds of reef complexes, whereas reduced connectivity is observed outside these complexes. This suggests that connectivity between reef complexes is relatively infrequent and does not occur at all for several reef combinations. Figure 2a also shows that virtual larvae released at the BA, INHC, and the MRC are generally swept southward. INHM is the only reef complex to exhibit bidirectional dispersal of virtual larvae both to the north and to the south.

BA reefs exhibit a high degree of within-complex connectivity. Figure 2a suggests that larvae released in the BA generally do not travel beyond the INHM reef complex. Furthermore, the connectivity between the BA and INHM weakened with increasing latitude. However, the connectivity matrix shows connectivity between the BA and the MRC, although with a low number of connected larvae. Additionally, a larger proportion of source reefs in the BA exhibit connectivity to the MRC compared to the geographically closer INHC reefs. This suggests that the mechanism for transporting larvae from the BA, the northernmost complex in this study, to southern reef complexes is not conducive to transporting larvae along the Delagoa Bight and results in the reefs at INHC being bypassed.

Reefs within the INHM complex showed relatively low levels of connectivity, but exported larvae to the destination reefs both to the north and south. The northern INHM reefs show stronger connectivity to the southern BA reefs; however, a similar pattern is not observed for the southern INHM reefs.

Source reefs at INHC only exhibit a southerly dispersal of virtual larvae, although they also indicate a reasonable level of self-recruitment. This implies that for the parameters used in this study, there is no mechanism that delivers larvae north across the Delagoa Bight.

MRC reefs deliver relatively few larvae to other reef complexes. Specific reefs from the MRC exhibit northerly dispersal of larvae to INHC, particularly those from the Northern Reef Complex. The Southern Reef Complex is a strong destination reef for larvae released throughout the MRC.

### Larvae travel times

Larvae released from the source reefs were transported to the destination reefs by regional currents that play a significant role in shaping connectivity within the region. The role of regional flow structures implies that specific reef pairs may be connected only during flow patterns with particular characteristics, potentially resulting in consistent travel times when these events occur. Figure 2b presents a matrix of mean and standard deviation travel times for all virtual larvae released from source reef *i* and arriving at destination reef *j*, indicating increased travel times with increasing distance from the source reef. The results also show that average and standard deviation travel times for within-complex connectivity are generally consistent throughout all reef complexes. Individual complexes are visually distinguishable, with average travel times of around 150 hours for within-reef connectivity. Compared to the BA and INHM complexes, average travel times within the MRC are somewhat lower. Given that reefs within the MRC are similarly spaced to those within northerly complexes, the shorter travel time suggests that dispersal within the MRC may be enhanced by the Agulhas Current.

Figure 2b provides greater context for the limited connectivity observed between INHM and the BA. Although these are neighbouring complexes, larvae travelling north from INHM generally required close to the full PLD of approximately 10 days to reach destination reefs. Additionally, the low travel time variability observed for this interaction was associated with northerly alongshore currents that precede the arrival of an anticyclonic eddy migrating southward from the Mozambican Channel. Similarly, connectivity between the BA and the MRC was associated with long-duration, low-variability travel times that likely also correspond to a distinct flow pattern. This interaction between the respective complexes is also infrequent, indicated by the low number of larvae reaching the destination.

The results from Figure 2b highlight the importance of regional flow structures in influencing the degree of connectivity of coral reefs in the study area. Flow structures are significant in determining the composition of incoming larval recruits at destination reefs; therefore, a sound understanding of these patterns will provide valuable information on the sources of biodiversity at downstream reefs in connected systems. These flow patterns are presented in detail in the following section.

### Temporal variability in connectivity

**Fig. 3:**
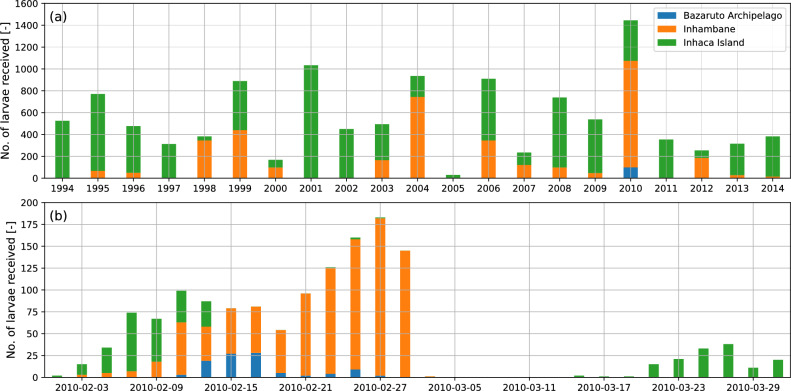
Stacked bar plot showing (**a**) the total number of larvae received by all reefs in the MRC from neighbouring reef complexes and (**b**) the total number of larvae received by the MRC for each individual 10 day simulation.

Coral reefs in the MRC were further assessed to calculate the temporal variability of neighbouring reef contributions. In addition to its marginal nature, the MRC was selected because of its primary role as a destination rather than a source. The total number of larvae received by all reefs within the MRC on all release days was summed for each simulated year; these results are presented in the upper panel of Figure 3. The lower panel of Figure 3 presents the total number of larvae received by the MRC for each release day in 2010. Larvae exchanged within the MRC have been intentionally omitted to emphasise the contribution of neighbouring reef complexes.

Figure 3 indicates a decreasing level of connectivity with increasing distance from the source reef complex to the MRC. INHC, the closest neighbouring reef complex, was continually connected to the MRC, although there were periods where the number of larvae arriving at the destination reef was relatively low, such as in 1998, 2005 and 2012. Connectivity to INHM was more sporadic and included several years of no connectivity during the simulated period.

In particular, connectivity between the BA and the MRC occurred exclusively in 2010. Connectivity from INHM was also significant during this period, which is plausible given the location of these reef complexes along the Southeast African coast. Temporal exchanges between the MRC and the neighbouring complexes, as shown in Figure 3b, indicate that connectivity between the MRC and INHC rarely occurred simultaneously with connectivity to the BA. Furthermore, the highest levels of larval exchange between INHM and the MRC occurred after the connectivity peak with the BA. These results highlight the variability in connectivity associated with variations in regional flow patterns.

### Regional flow patterns

**Fig. 4:**
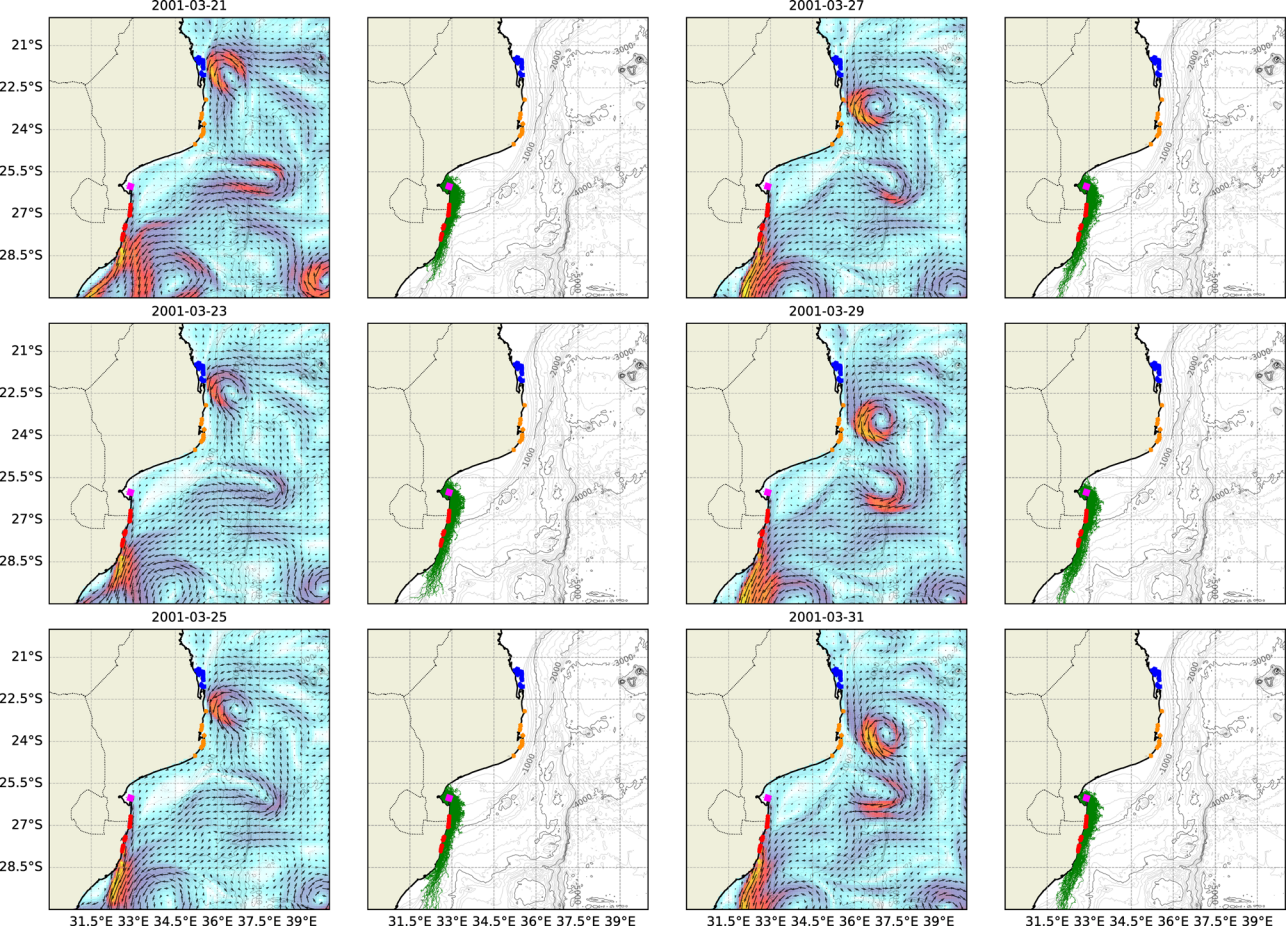
Instantaneous near-surface currents and larval tracks for a regional flow pattern resulting in significant connectivity between coral reefs at INHC and the MRC. Individual reef complexes including the BA (*blue*), INHM (*orange*), INHC (*magenta*) and the MRC (*red*) are also shown.

**Fig. 5:**
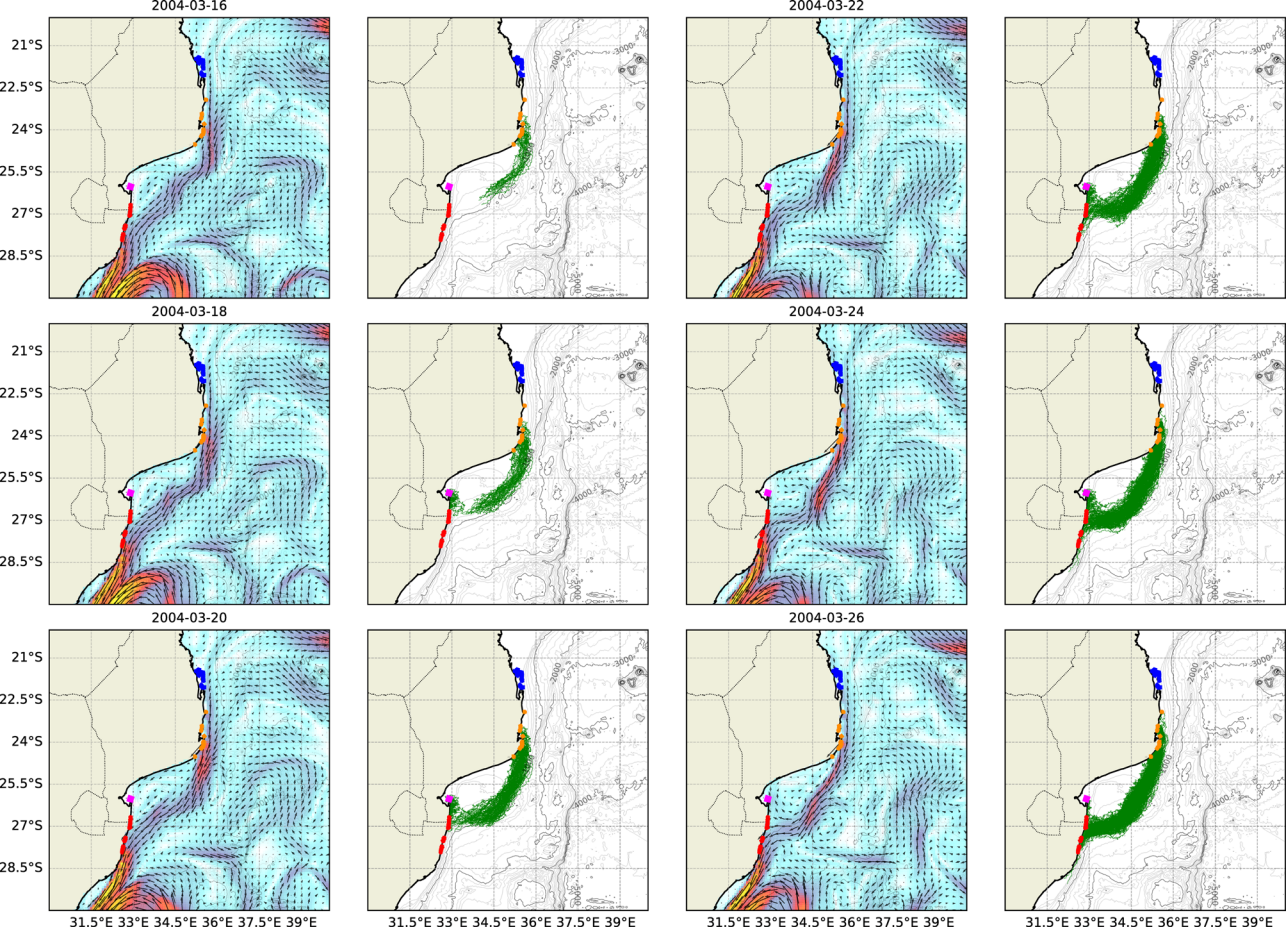
Instantaneous near-surface currents and larval tracks for a regional flow pattern resulting in significant connectivity between coral reefs at INHM and the MRC. Individual reef complexes including the BA (*blue*), INHM (*orange*), INHC (*magenta*) and the MRC (*red*) are also shown.

**Fig. 6:**
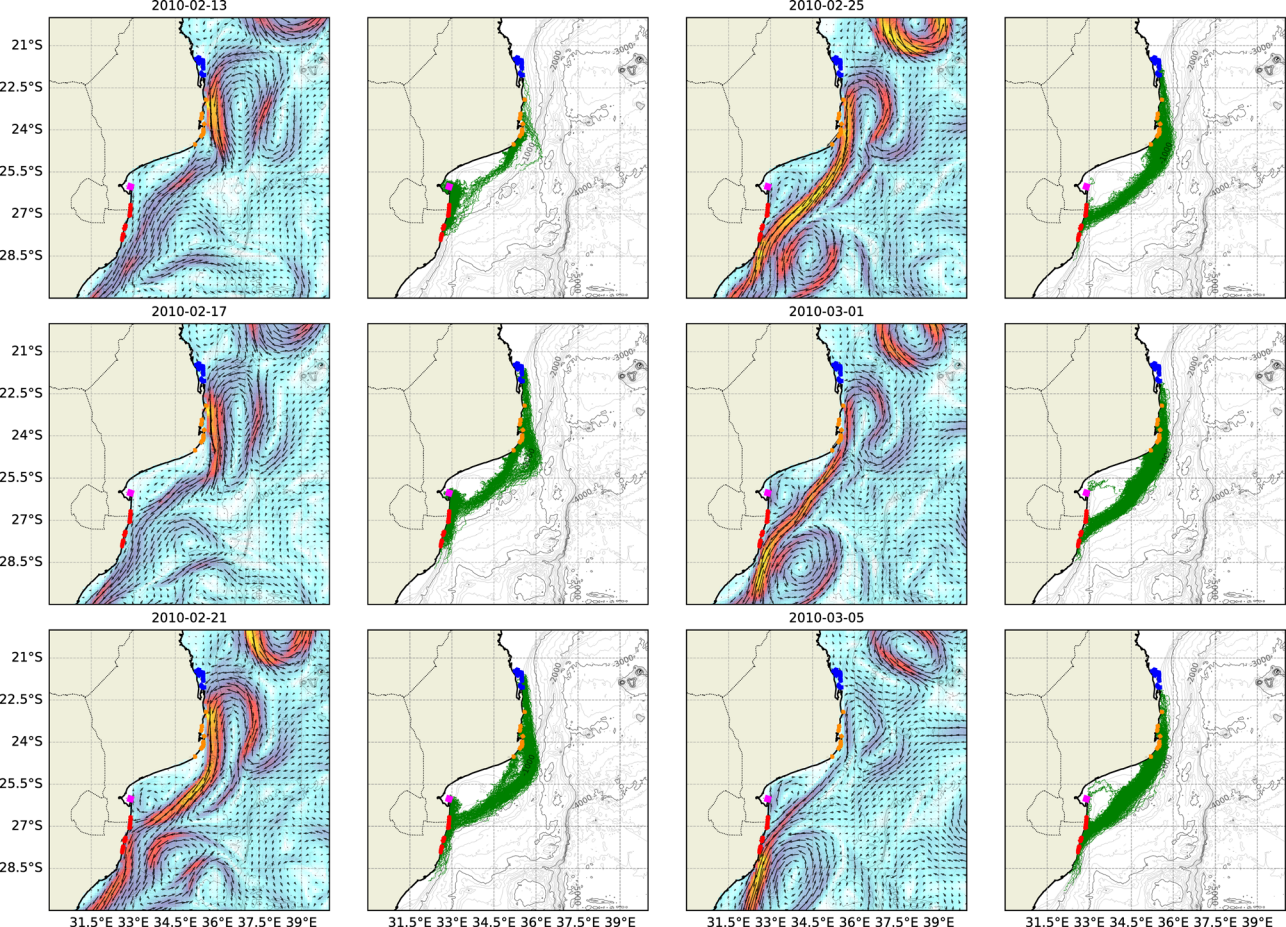
Instantaneous near-surface currents and larval tracks for a regional flow pattern resulting in significant connectivity between coral reefs at the BA and the MRC. Individual reef complexes including the BA (*blue*), INHM (*orange*), INHC (*magenta*) and the MRC (*red*) are also shown.

Regional flow patterns that resulted in significant larval contributions from neighbouring complexes are presented in Figures 4, 5 and 6. Each figure presents the relevant flow pattern through multiple instantaneous snapshots alongside the associated tracks for larvae that arrived at destination reefs. Tracks of virtual larvae within their competency window were considered for all simulations that overlapped with the snapshot date. In addition, larval tracks are presented for the labelled date for each subplot. This implies that larvae reaching the MRC towards the latter stages of the regional flow pattern appear as seemingly incomplete paths. However, these paths were shown to get progressively closer to the destination over time.

#### Inhaca Island

Figure 4 presents instantaneous snapshots of the regional flow pattern associated with high levels of connectivity between coral reefs in INHC and the MRC. The pathways travelled by larvae originating from INHC that reached the MRC are also presented.

The regional flow pattern in Figure 4 shows that connectivity between these reef complexes was associated with the occurrence of a stream along the Delagoa Bight. The stream originated near the INHM reefs and travelled alongshore, eventually merging with the Agulhas Current near the southern MRC. Alongshore streams in the Delagoa Bight occurred as a result of the deflection of currents around the coastline undulation at INHM. This significant coastline feature, together with slower currents in the region, allowed these alongshore flows to form and persist. Notably, connectivity was observed only between the INHC and MRC complexes for this slow pattern. The slow speed of the stream along the Delagoa Bight prevented larvae from the INHM complex from reaching the MRC complex within their competency window.

#### Inhambane

Figure 5 presents instantaneous snapshots of the regional flow pattern associated with high levels of connectivity between coral reefs at INHM and the MRC, showing the pathways travelled by larvae between these two complexes.

Figure 5 indicates that strong connectivity between INHM and the MRC was associated with a high-velocity stream originating in the vicinity of the source reefs. The stream provided a direct pathway between the reef complexes. Similar to the flow pattern for INHC, the INHM coastline played a significant role in influencing currents in the region. A marked difference from the flow pattern connecting INHC to the MRC (Figure 4) was the formation of a cyclonic eddy along the Delagoa Bight resulting from the shadow zone cast by the stream between INHM and the MRC. As a result, connectivity between INHC and the MRC was prevented by increased travel times that exceed the larval competency window used in this study. Furthermore, the flow patterns in Figure 5 prevented connectivity between the BA and the MRC due to slower currents north of Inhambane.

#### Bazaruto Archipelago

Figure 6 presents instantaneous snapshots of the regional flow pattern associated with high levels of connectivity between coral reefs in the BA and MRC, showing the pathways travelled by larvae between these two complexes.

Figure 6 presents a flow pattern similar to that in Figure 5, showing the formation of a concentrated, high-velocity stream connecting the BA to the MRC. The flow pattern in Figure 6, however, was notably faster and originated further north, allowing larvae released at the BA to reach the MRC within their competency window. Figure 6 also shows the progressive formation of a cyclonic eddy along the Delagoa Bight, which appeared to reduce the degree of connectivity between INHC and the MRC over time. The high-velocity stream, formed on 25/02/2010, followed the interaction of a large anticyclonic eddy with the Southeast African coastline. Furthermore, Figure 6 also highlights the presence of additional anticyclonic eddies in the south of the model domain. The collective presence of these eddies acted to direct the high-velocity stream shoreward, resulting in the high degree of connectivity observed between these reef complexes. This contrasts with the flow patterns in Figure 5, where the flow structure did not appear associated with large-scale regional features.

## Discussion

Lagrangian particle tracking of virtual larvae representing the broadcast spawning species *A. austera* with a PLD of 10 days provided insights into the extent of connectivity between coral reefs along the Southeast African coastline. Results indicated notable connectivity between neighbouring reefs within the domain along with significant levels of self-recruitment at all reef complexes. Reef connectivity was driven by distinct regional flow patterns associated with variations of a generally southerly current that connected coral reefs. Stronger, concentrated southerly currents favoured connectivity between distant reefs, while weaker southerly currents resulted in flow reversals along the Delagoa Bight due to cyclonic eddies. Analysis of travel times for connectivity events between reef pairs suggested that infrequent connectivity generally yielded low variability, alluding to a dominant hydrodynamic association between the pair. Assessment of regional-scale currents during connectivity peaks between reef complexes showed that the coastline configuration, particularly the Delagoa Bight, influenced regional currents that were associated with high levels of connectivity. A strong southward stream originating at INHM connected northerly reefs and the MRC. This resulted in relatively short travel times for larvae compared to their PLDs. Comparatively, connectivity between INHC and the MRC was associated with a weaker current stream along the Delagoa Bight, resulting in lengthy travel times, often exceeding the PLD.

Our connectivity results showed that the MRC received the highest number of larvae from neighbouring complexes in 2010. Porter and Schleyer^12^ observed a notable increase in hard coral recruitment in 2010 during their study on the long-term dynamics of coral reefs in Sodwana Bay. Their findings indicated an increase to around 100 recruited colonies from 20 in the previous year. This peak was followed by a return to typical recruitment levels, such as those observed in 2009. Results also show that the greatest contributor to the MRC in 2010 was INHM, followed by INHC; however, 2010 also represented the only year that the BA connected to the MRC, although to a lesser extent. An earlier study on coral recruitment at numerous reefs within the MRC between 2000 and 2002 by Glassom^21^ found that the majority of Acroporidae recruitment occurred during a single event in March 2001, with minimal recruitment measured in 2000 and 2002. Schleyer et al.^56^ conducted a similar study measuring coral recruitment and mortality at Nine-Mile Reef between 1993 and 2006. Their findings showed a decreasing trend of hard coral recruitment over the measurement period. Both studies were carried out during periods of consistent connectivity between the MRC and the neighbouring INHC and INHM complexes in our simulations, suggesting that the increase in recruitment observed in 2010 was potentially the result of larvae arriving from these two sources. This study, however, did not account for the magnitude of larvae spawned at source reefs due to insufficient data. Therefore, there remains some uncertainty around the contributions of individual reef complexes to the MRC. Biophysical factors such as variable mortality and post-settlement survival were not considered but may strongly influence connectivity between reefs. Connolly and Baird^57^ studied the competency acquisition rate in several coral species, finding that competence peaked relatively quickly, followed by a prolonged exponential decline. The same authors also found that the mortality rate of the studied corals generally decreased exponentially with age. These findings concerning vital rates/population dynamics may significantly influence connectivity, particularly for events occurring towards the end of the species’ PLD.

Montoya-Maya et al.^17^ studied the evolutionary (genetic) connectivity between coral reefs in the western Indian Ocean for the broadcast spawning species *A. austera*, defined as “*the amount of gene flow between populations over a timescale of several generations*”. Their findings suggested that South African coral populations were independent of gene flow from northern coral populations on ecological time scales. Furthermore, their study provided evidence that coral reefs in this region were connected at evolutionary time scales, involving gene flow between populations over the timescale of multiple generations. This finding is supported by the results of our study, which showed that connectivity between the MRC and the BA was highly infrequent and required specific regional flow patterns. This suggests that regional hydrodynamics regulate evolutionary connectivity at the MRC. Montoya-Maya et al’s^17^ study also indicated that the number of migrants per generation arriving at the BA from the MRC was nearly half that arriving at the MRC from INHC. Macdonald et al.^15^ reported similar findings in their study on recruitment at Sodwana Bay, asserting that individual coral complexes should be managed separately rather than as one interconnected population. The results of this study support this assertion, showing that recruitment events linked to the influx of larvae from distant reefs are potentially infrequent and may be negatively affected by mass mortality events at the source reefs. McManus et al.^58^ found that evolution was a critical factor in preventing extinction while enabling the long-term recovery of coral reef communities in their study. High-latitude reefs that rely on low-latitude reefs as sources of evolutionary connectivity are at high risk of losing this supply given the vulnerability of low-latitude coral reefs to future thermal stresses^18^.

Anticyclonic mesoscale eddies in the north of the domain were a prominent feature in Figures 4, 5 and 6, which detail the regional flow structures that are associated with connectivity between reef complexes. These features resulted in strong southerly streams due to western intensification along the coastline. The eddies are generated in the Mozambican Channel, an area of high mesoscale kinetic energy^14^. Huang^59^ found that these anticyclonic eddies were predominantly generated within the Comoros Basin due to barotropic instabilities. This generation mechanism was also suggested by Biastoch and Kraus^60^ and Roberts et al.^61^. A notable proportion of anticyclonic eddies (86%) and cyclonic eddies (77%) that can traverse the narrowest section of the channel develop into dipoles^59^, as shown in Figure 4. The high current speeds within the stream occurred within a narrow region, resulting in the formation of cyclonic eddies along the Delagoa Bight due to this region lying in the lee of the Delagoa Peninsula. The streams form part of the Agulhas Current, an intensified western boundary current^62,63^. Biastoch et al.^63^ state that the Agulhas Current has a reach of approximately 1500 m in depth with the highest current speeds occurring at the surface. The duration of the streams that form at the Delagoa Peninsula is likely linked to the time scale of the eddies that migrate from the Mozambique Channel. Huang et al.^59^ estimated that dipole eddy time scales in the Channel typically do not exceed 10 days, although several eddies have been observed with time scales longer than 30 days. This is a potentially crucial limiting factor for the dispersal of short PLD larvae from northerly reefs to southerly reefs in the study region. Additionally, Yang et al.^62^ suggested that the Agulhas Current showed a trend of southward migration, potentially resulting in reduced flow speeds around the Delagoa Bight. Wells et al.^64^ found that the strong, intermittent southward stream was associated with upwelling along the Delagoa Peninsula that is subsequently advected to the Sodwana Bay reefs. The complex dynamics of the WIO are a significant factor for the long-term sustainability of high-latitude reefs in the WIO.

Reanalysed global ocean currents are an ideal data source for performing regional-scale studies; however, they have limitations in spatial and temporal resolution. This limits the inclusion, within particle tracking simulations, of complex, fine-scale hydrodynamic features that may provide greater insights. Future work will focus on high-resolution simulations of currents in the region for detailed particle tracking investigations. Furthermore, this study focused on physical connectivity and did not include biophysical larval traits such as mortality and autonomous movement. Future work aims to include more advanced larval dispersal models that integrate these parameters, such as that used by Burt et al.^25^, to provide a more comprehensive estimation of coral reef connectivity in the region.

## Conclusion

This study investigated the potential connectivity between coral reef complexes in the WIO for a short PLD species of hard coral between 1994 and 2014 using Lagrangian particle tracking with reanalysed modelled global ocean currents. Results showed that connectivity between distant reef complexes was generally associated with specific regional currents that facilitated larval transport within their PLD. Furthermore, these regional flow patterns interacted with the Southeast African coastline and were associated with high-velocity streams and cyclonic eddies offshore of and along the Delagoa Bight respectively. Results also suggest that evolutionary connectivity that has been identified between Mozambican and South African reefs is potentially regulated by the regional hydrodynamics, particularly for coral species with short larval durations. Connectivity between northerly Mozambican reefs and the MRC was associated with high-speed current streams originating at the Delagoa Peninsula. These streams are part of the greater Agulhas Current; however, they are generally short-duration events due to the intermittency of eddies propagating south from the Mozambique Channel. These streams commonly resulted in the formation of a cyclonic eddy along the Delagoa Bight that entrained larvae released at INHC, leading to travel times beyond their PLD. The shape of the Southeast African coastline plays a significant role in regulating the connectivity of coral reefs in the region by influencing the formation of regional-scale currents. The complex circulation in this region is therefore a significant factor for the long-term sustainability of high-latitude reefs in the WIO and needs to be considered in research and conservation programs. This study, however, was undertaken using reanalysed global ocean currents at a relatively coarse scale and therefore does not resolve complex fine-scale flow structures formed along the coastline. Therefore, a more comprehensive study on the local hydrodynamics of the MRC reefs is recommended to understand these processes in greater detail.

## Data Availability

Lagrangian particle tracking simulation outputs and the corresponding processing scripts are available upon request from the corresponding author, Vibhav Deoraj.
